# Andrographolide and Deoxyandrographolide Inhibit Protease and IFN-Antagonist Activities of Foot-and-Mouth Disease Virus 3C^pro^

**DOI:** 10.3390/ani12151995

**Published:** 2022-08-07

**Authors:** Sirin Theerawatanasirikul, Varanya Lueangaramkul, Nattarat Thangthamniyom, Penpitcha Chankeeree, Ploypailin Semkum, Porntippa Lekcharoensuk

**Affiliations:** 1 Department of Anatomy, Faculty of Veterinary Medicine, Kasetsart University, Bangkok 10900, Thailand; 2 Department of Microbiology and Immunology, Faculty of Veterinary Medicine, Kasetsart University, Bangkok 10900, Thailand; 3 Center for Advanced Studies in Agriculture and Food, Kasetsart University Institute for Advanced Studies, Kasetsart University, Bangkok 10900, Thailand

**Keywords:** 3C protease (3C^pro^), andrographolide, antiviral activity, deoxyandrographolide, diterpenoids, foot-and-mouth disease virus, intracellular protease inhibitory assay, molecular docking

## Abstract

**Simple Summary:**

Foot-and-Mouth disease (FMD) is a re-emerging infectious disease that poses a negative impact on livestock production and economics worldwide. It is also endemic in underdeveloped and developing countries, mostly in tropical areas. The control of this highly contagious disease requires a combination of different strategies, including the culling of infected animals, reducing animal movement, and vaccination. Although vaccination is effective, there remains a non-protective interval after immunization. Antiviral agents that can inhibit FMD virus (FMDV) could reduce the shedding of viruses in terms of quantity and duration, which could assist other control measures to contain FMD spreading. Antiviral activities of plant-based products, including andrographolides, have been demonstrated in several studies. Andrographolides are a group of phytochemical compounds derived from medicinal plants in the genus *Andrographis*, which are abundant in Asia, a hot spot of FMDV outbreaks. We found that andrographolides could inhibit FMDV replication by targeting a viral protease, namely 3C^pro^. FMDV 3C^pro^ is the main protease essential for the virus life cycle. The 3C^pro^ also counteracts type I interferon, which is the frontline antiviral cytokine. We also revealed the intracellular mechanisms by which the andrographolides inhibited both protease and IFN antagonist activities of the 3C^pro^.

**Abstract:**

Foot-and mouth-disease (FMD) caused by the FMD virus (FMDV) is highly contagious and negatively affects livestock worldwide. The control of the disease requires a combination of measures, including vaccination; however, there is no specific treatment available. Several studies have shown that plant-derived products with antiviral properties were effective on viral diseases. Herein, antiviral activities of andrographolide (AGL), deoxyandrographolide (DAG), and neoandrographolide (NEO) against FMDV serotype A were investigated using an in vitro cell-based assay. The results showed that AGL and DAG inhibited FMDV in BHK-21 cells. The inhibitory effects of AGL and DAG were evaluated by RT-qPCR and exhibited EC50 values of 52.18 ± 0.01 µM (SI = 2.23) and 36.47 ± 0.07 µM (SI = 9.22), respectively. The intracellular protease assay revealed that AGL and DAG inhibited FMDV 3C^pro^ with IC50 of 67.43 ± 0.81 and 25.58 ± 1.41 µM, respectively. Additionally, AGL and DAG significantly interfered with interferon (IFN) antagonist activity of the 3C^pro^ by derepressing interferon-stimulating gene (ISGs) expression. The molecular docking confirmed that the andrographolides preferentially interacted with the 3C^pro^ active site. However, NEO had no antiviral effect in any of the assays. Conclusively, AGL and DAG inhibited FMDV serotype A by interacting with the 3C^pro^ and hindered its protease and IFN antagonist activities.

## 1. Introduction

The foot-and-mouth disease virus (FMDV) causes a highly contagious disease in cattle, pigs, sheep, goats, buffalo, and other cloven-hoofed wildlife animals [1]. Currently, the FMD Quarterly has reported the global outbreaks of six different serotypes of FMDV –O, A, Asia-1, and Southern African Territories (SAT) 1, 2, and 3, which still actively spread and circulate in many countries worldwide [2]. FMDV serotype C has disappeared since 2004, the last report was from Kenya [3]. Recently, the outbreaks within the endemic pools have been updated in Thailand, Malaysia, Vietnam, and some countries in southern Africa [2]. Animals with FMD show vesicles in and around the mouth, interdigital spaces, and feet. Once the vesicles erupt, the viruses in the vesicular fluids can spread to non- or partial-immune animals. In addition, FMDV may persist in some animals and transmit to the naïve animals in the herd resulting in a long-term circulation of the virus on the farm, which is the major course of the production loss [4].

FMDV belongs to the genus *Aphthovirus* of the family *Picornaviridae* [3,5], which is a positive-sense, single-stranded RNA virus. The 7 kb genomic RNA contains a single open reading frame (ORF) coding for a polyprotein, which is flanked by a long 5′-untranslated region (UTR) and a short 3′-UTR with a poly (A) tail [6,7]. The virus encodes three proteases, including leader (L^pro^), 2A, and 3C^pro^. Once translated, the L^pro^ cleaves itself from the remaining polyprotein. The 2A protease is a self-cleavage peptide that excises itself immediately after translation resulting in a precursor protein P1-2A. The 3C^pro^ cut between the P2 and P3 precursors and processes the polyproteins into mature structural (VP1–VP4) and non-structural proteins (NSPs) that function in the viral life cycle [1,8].

In the endemic area where culling is problematic, vaccinating livestock is the major FMDV control strategy [1]. However, FMDV has continually evolved, even in vaccinated animals, especially when the virus vaccine does not perfectly match the field strain. This causes the emergence and circulation of the escape mutants in the animal populations. When vaccination does not prevent the infection or vaccination is delayed, widespread and long-term outbreaks of FMD may occur because the infected animals can shed the virus to non- or partial-immune animals [4]. Thus, antiviral agents that can reduce the level and duration of virus shedding hold the potential to have a positive effect on the FMDV control program. Currently, plant-derived antiviral agents are widely used because natural products are cheap and available in the local areas. Some plants have low toxicity and can prevent microbial infections [9]. Recently, more evidence has shown that plant-based products could be an alternative therapeutic agent for combating some viral infection by accelerating the disease recovery period or protecting animals during the time lag between vaccination and immunity [10].

Andrographolide is a diterpenoid lactone, which is a bioactive substance derived from the aerial part of plants in the genus *Andrographis* (family *Acanthaceae*). Particularly, *Andrographolide paniculata* is a medicinal plant found in most Asian countries and has been used in traditional Chinese and Thai medicines [11]. The secondary metabolites of *A. paniculata* and andrographolide derivatives possess therapeutic properties, such as antiviral, antimicrobial, and anti-parasitic activities, and biological effects including antioxidant and anti-inflammation (reviewed by [12,13]). The main bioactive substances of *A. paniculata* are diterpenes that contain a γ-lactone ring found in andrographolide (AGL), deoxyandrographolide (DAG), neoandrographolide (NEO), and a few of didehydroandrographolides (DDAG). AGL and DAG are mostly present in the leaves of *A. paniculata* [14]. Among the main phytoconstituents of *A. paniculata*, antiviral activities of AGL have been reported at substantial levels against various viruses, including enteroviruses-D68 [15], influenza A virus [16], hepatitis B virus [17], hepatitis C virus [18], herpes simplex virus I [19], human immunodeficiency virus [20], chikungunya virus [21], and severe acute respiratory syndrome coronavirus 2 (SARS-CoV-2) [22]. However, how AGL and its derivatives inhibit the viruses and their target molecules is still unknown.

In this study, we investigated the antiviral activities of the three major bioactive substances from *A. paniculata* using an in vitro cell-based assay. Both AGL and DAG but not NEO inhibited FMDV replications. Furthermore, our intracellular protease assay and molecular docking revealed that the phytochemical compounds inhibited FMDV by interacting with 3C^pro^, which in turn inactivated the protease and alleviated its IFN-antagonist activity.

## 2. Materials and Methods

### 2.1. Cells and Virus

The baby hamster kidney cell line (BHK-21; ATTC^®^, Manassas, VA, USA) and the human embryonic kidney 293T cell line (HEK 293T; ATCC^®^) were cultured in minimum essential medium (MEM; Gibco^TM^ Thermo Fisher Scientific Inc., Waltham, MA, USA) for the antiviral activity assay and Opti-MEM (Gibco^TM^ Thermo Fisher Scientific Inc., Waltham, MA, USA) for the transfection experiments, respectively. Both media were supplemented with 10% (*v*/*v*) fetal bovine serum (FBS; Gibco^TM^ Thermo Fisher Scientific Inc., Waltham, MA, USA), and all cell cultures were incubated at 37 °C with 5% CO_2_. FMDV serotype A, A/TAI/NP05/2016 (NP05) was propagated in BHK-21 cells as previously described [23] and stored at −80 °C until used. The BHK-21 and HEK 293T cells (10^5^ cells/mL) were seeded in each well of a 96-well plate for viral infection and transfection, respectively.

### 2.2. Andrographolides

AGL, DAG, and NEO with ≥98% purity were purchased from ChemFace (Wuhan, China); code: CFN98923, CFN90518, and CFN97766, respectively). The andrographolides were dissolved in 100% dimethyl sulfoxide (DMSO) to the final stocks of 10 mM and stored at −80 °C until used. The andrographolides were serially diluted to various concentrations in MEM supplemented with 2% FBS (maintenance media), and the final concentration of DMSO in each well was less than 0.01%.

### 2.3. Cytotoxicity Assay

The BHK-21 and HEK 293T cells were seeded in 96-well-tissue culture plates until the cell monolayer reached approximately 90% confluence on the next day. The andrographolides were freshly diluted in complete media to the final concentrations of 0.1, 1, 5, 10, 25, 50, 75, 100, 200, and 250 µM. The cell culture media was removed and replaced with various concentrations of andrographolides in duplicates. The cells incubated with media alone or media with 0.01% DMSO were included as negative controls. The andrographolide-treated and control cells were incubated at 37 °C for 24 h. Viable cells were determined using CellTiter 96^TM^ Aqueous non-radioactive cell proliferation assay, a colorimetric method (MTS assay, Promega, Madison, WI, USA) as previously described [24]. Briefly, the color was developed depending on cell viability, and the optical density (OD) in each well was measured at the wavelength of 490 nm. The ODs of the drug treatment wells were compared to the well with 0.01% DMSO using the following equation:

$$\frac{\left[\mathrm{OD}\;\mathrm{treated}\;–\mathrm{OD}\;\mathrm{cell}\;\mathrm{control}\right]}{\left[\mathrm{OD}\;0.01\%\;\mathrm{dmso}-\mathrm{OD}\;\mathrm{cell}\;\mathrm{control}\right]}$$

The 10% and 50% cytotoxicity concentrations (CC10 and CC50) were analyzed using a fitting curve of non-linear regression (GraphPad version 9.0, Prism; San Diego, CA, USA). The CC10 value is the maximum, non-toxic concentration of a compound that causes 10% cell death; thus, at least 90% of viable cells remain in the test unit. On the other hand, the CC50 would be used in the formulation present in Section 2.4.

### 2.4. Antiviral Activities of the Andrographolides

Antiviral activity assays were performed as described previously with some modifications [25]. Briefly, the antiviral activities of andrographolides were evaluated at various times of infection, including before and during viral absorption and post-infection. BHK-21 cells were seeded into 96-well cell culture plates and incubated until the cell monolayers reached 80% to 90% confluent. The cell culture medium was removed, and the wells were washed with phosphate-buffered saline) pH7.4 (PBS, Invitrogen, Carlsbad, CA, USA). In the post-infection assay, FMDV was diluted to the final concentration of 10 median tissue culture infectious doses (TCID50) before incubating with the cells at 37 °C for 2 h. Subsequently, the inoculum was removed, and the infected cells were washed three times with PBS, followed by the addition of the freshly prepared AGL, DAG, and NEO in the complete media at various concentrations (at 1, 10, 25, 50, 100, and 150 µM) according to the CC50 values. In the viral absorption assay, the diluted andrographolides and virus were added simultaneously onto the cells and incubated at 37 °C for 2 h. For the viral protection assay, the BHK-21 cells were incubated with various dilutions of the andrographolides for 2 h prior to incubation with FMDV for 2 h. In the viral absorption and protection assays, after finalized incubations, the supernatants were removed and replaced with the maintenance media. The andrographolides treated cells were incubated at 37 °C for 24 h. The infected cells incubated with 0.01% DMSO alone and non-infected BHK-21 cells were included as the vehicle and virus-negative controls, respectively. Each treatment was run in duplicate wells. All assays were performed independently in triplicates.

### 2.5. Immunoperoxidase Monolayer Assay (IPMA)

The mock- and FMDV-infected BHK-21 cells incubated with 0.01% DMSO alone as a vehicle control or with the andrographolides obtained from the antiviral activity assay were fixed with cold absolute methanol for 15 min and examined using the IPMA assay as previously described [26]. Briefly, the fixed cells were incubated with 0.1% H_2_O_2_ in distilled water for 30 min and then rinsed using 0.1% tween 20 in PBS (PBST) before incubation with 3% bovine serum albumin in PBS for 30 min. The viral infection was incubated with a single-chain variable fragment with Fc fusion protein (scFv-Fc) specific to 3ABC of FMDV [25] at 37 °C for 1 h. The cells were washed twice with PBST and subsequently incubated with HRP-conjugated protein G (EMD Millipore Corporation, Temecula, CA, USA) at 37 °C for 1 h. The antigen–antibody complex was detected by DAB substrate, which appeared with a dark-brown color in the FMDV-infected cells. The cells were observed under a phase-contrast inverted microscope (Olympus IX73, Tokyo, Japan), and all images were recorded prior to counting the numbers of the viral infected cells by using CellProfiler version 4.1.3 (Board Institute, Cambridge, MA, USA) as previously described [24,25]. Antiviral activity was evaluated and reported as 50% effective concentrations (EC50) using non-linear regression (GraphPad version 9.0, Prism, USA). The EC50 is the drug concentration that can inhibit half of the viruses used in the assay. The selective index (SI) was calculated by dividing the CC50 by EC50, as shown in the following equation.

$$\mathrm{SI}\;=\frac{\mathrm{CC}50}{\mathrm{EC}50}$$

### 2.6. FMDV RNA Quantification Using RT-qPCR

The BHK-21 cells were seeded onto 24-well cell culture plates and incubated at 37°C with 5% CO_2_ overnight or until the cell monolayer reached 80% to 90% confluence. The cells were inoculated with FMDV at 10 TCID50 and incubated at 37 °C with 5% CO_2_ for 2 h. Subsequently, the inoculum was removed and replaced with the andrographolides at various concentrations (25, 50, 100, and 150 µM). The cells were further incubated at 37 °C for 24 h. Mock-infected cells and DMSO treatment cells were included as viral- and vehicle-negative controls, respectively. The cells and supernatant were collected from each of the culture wells for viral load quantification. Briefly, total RNAs were extracted using Trizol^TM^ reagent (Thermo Fisher Scientific Inc., USA) and then clarified using Direct-zol MiniPrep (Zymo Research Corporation, Tustin, CA, USA). The RNA quality was evaluated using Qubit™ RNA high sensitivity (HS) assay kit, and the solution mixtures were measured by Qubit^TM^ 4 fluorometer (Thermo Fisher Scientific Inc., Waltham, MA, USA) according to the manufacturer’s instruction. One microgram of the total RNA was used as the template for cDNA synthesis using random hexamers and RevertAid reverse transcriptase enzyme (Thermo Fisher Scientific Inc., Waltham, MA, USA) as previously described [25]. Then, 2 µL of the cDNA were used for qPCR amplification with primers specific to the FMDV 5′UTR, fwd primer: 5′–CTGTTGCTTCGTAGCGGAGC–3, and rev primer: 5′–TCGCGTGTTACCTCGGGGTACC–3′, as previously described [23,25]. A standard curve of viral copy numbers was generated by ten-fold serially diluting the plasmid pFMDV 5′ UTR [25] from 10^−1^ to 10^−7^ plasmid molecules/μL. The qPCR amplifications were performed using SsoFast EvaGreen Supermix (Bio-Rad Laboratories, Hercule, CA, USA) in the C1000 Touch thermal cycler (Bio-Rad Laboratories, USA). The cycle conditions were an initial DNA denaturation at 95 °C for 30 s and 30 cycles of denaturation at 95 °C for 5 s and annealing plus extension at 60 °C for 5 s, followed by a melting curve analysis from 65 to 95 °C with a 0.5 °C increment as described previously [25]. In each experiment, three biological replicates were performed independently, and technical duplicate wells were run in the real-time PCR. The percentages of viral reductions were calculated as viral copy numbers in the treatment wells relative to that of 0.01% DMSO control.

### 2.7. Intracellular Protease Inhibitory Assay

To determine the effect of andrographolides on the FMDV 3C^pro^ activity, an intracellular protease assay was performed as described elsewhere [26]. Briefly, 100 µL of 1 × 10^3^ HEK 293T cells in Opti-MEM (GibcoTM Thermo Fisher Scientific Inc., Waltham, MA, USA) were cultured in each well of a 96-well cell culture plate and incubated at 37 °C until they reached 80% to 90% monolayers on the transfection day. The plasmids used in this assay are pBV_3ABCD, pBV_mu3ABCD, and pG5Luc [25,27,28]. pBV_3ABCD contains FMDV 3ABCD ORF encoding an intact 3C protease inserted between the Gal4-binding domain and the VP16-activation domain of plasmid pBV [25]. pBV_mu3ABCD was constructed similarly to pBV_3ABCD; however, the 3C^pro^ ORF was mutated at two amino acid residues, Cys142Ser and Cys163Gly, to destroy the protease activity [27,28] and served as the protease-negative control. The pG5Luc composes of the GAL4 binding site upstream VP16 and the firefly luciferase reporter gene sequences, followed by the *Renilla* luciferase gene. When co-transfection of pBV_mu3ABCD and pG5Luc, GAL4 binds to the Gal4-binding domain, and the VP16-activation domain activates VP16 to drive the expression of firefly and *Renilla* luciferase. The *Renilla* luciferase served as an internal luciferase control in this detection system. However, upon translation, the 3C^pro^ expressed from pBV_3ABCD would cut at the 3A/B, 3B/C, and 3C/D junctions, which separated the Gal4-binding domain and VP16-activation domain, resulting in no firefly luciferase expression.

To perform the protease assay, the cells in each well were co-transfected with the mixture of 0.1 µg of either pBV_3ABCD or pBV_mu3ABCD, 0.1 µg of pG5luc plasmid, and 0.6 μL Fugene^®^ HD (Promega, Madison, WI, USA) in 10 µL of Opti-MEM. Each treatment and control were run in duplicate. The transfected cells were incubated at 37 °C for 2 h before adding various concentrations of the andrographolides previously prepared by diluting in Opti-MEM. The controls for this experiment included the vehicle control (0.01% DMSO, mock-treated (no drug) and non-transfected cells), empty plasmid control (mock-treated, cells transfected with pBV), 3C^pro^ positive control (0.01% DMSO, mock-treated (no drug) and cells transfected with pBV_mu3ABCD), protease inhibitor positive control (Rupintrivir treated, cells transfected with pBV_3ABCD), and 3C^pro^ negative control (mock-treated, cells transfected with pBV_mu3ABCD). At 16 h post-transfection, the cells were washed once with PBS and then lysed with 20 µL passive lysis buffer (Promega, Madison, WI, USA). To quantify the anti-3C^pro^ activity of the andrographolides, the firefly and *Renilla* luminescence signals were measured using Dual-Glo Luciferase Assay System (Promega, Madison, WI, USA) in Synergy H1 Hybrid Multi-Mode Microplate Reader (BioTek, Winooski, VT, USA). In this assay, the protease activity was reported as an inverse correlation of the firefly/*Renilla* luminescent (Fluc/Rluc) signal ratio from each compound treatment well in relation to the Fluc/Rluc ratio from no drug control. The 50% and 90% inhibitory concentration values (IC50 and IC90) were compound concentrations that increased the Fluc/Rluc signal ratios by 50% and 90%, respectively, compared with the 0.01% DMSO control.

### 2.8. Optimization of Interferon (IFN) β Treatment in HEK 293T Cells

To explore a suitable timepoint for the IFN-stimulated ISG transcripts, the HEK 293T cells were incubated with 1000 IU/mL IFN β, and the IFN β-treated cells were then harvested at 0, 2, 4, 8, 12, and 24 h after IFN β treatment. The ISG mRNA expression levels of IFN-stimulated gene 15 (ISG15), ISG56, 2′,5′-oligoadenylate synthetase (OAS-1), myxovirus resistance 1 (Mx-1), and double-stranded RNA-dependent protein kinase R (PKR) were initially up-regulated and significantly increased at high levels at 8 h as determined by RT-qPCR (Figure S1). Therefore, in the following experiment, the IFN-activated ISG expressions were evaluated at 8 h after the IFN β treatment.

### 2.9. Evaluating Effects of Andrographolides on the Inhibition of Interferon Stimulating Gene (ISG) Expressions by IFN-Antagonist Activity of 3C^pro^

To determine whether the compounds would indirectly derepress the IFN antagonist activity of the 3C^pro^ by increasing the levels of ISG mRNA expression, 1 × 10^4^ HEK 293T cells were seeded in each well of the 24-well cell cultured plates and incubated until the cell monolayers reached 80% to 90% confluent on the transfection day. The cells were transfected with the mixtures of 0.5 µg of pBV_3ABCD and 1.5 μL Fugene^®^ HD (Promega, Madison, WI, USA) before incubating at 37 °C for 2 h. Subsequently, the andrographolides at the doses of IC50 and IC90 were added to the transfected cells. The maximum, non-toxic concentration (CC10 values) was used as an alternative when IC90 was higher than the CC10. Additionally, empty vector pBV and pBV_mu3ABCD transfected cells were included for all treatments as plasmid background and 3C^pro^ negative controls, respectively. Mock-treated, non-transfected cells with IFN β treatment were added in this experiment as the IFN control to demonstrate the full spectrum of IFN activity. Each treatment and control were run in duplicates. After 24 h post-transfection, the cells were treated with 1000 IU/mL of IFN β (R&D system, Minneapolis, MN, USA) for 8 h. The total RNAs were isolated from the transfected and control cells using Trizol^TM^ reagent (Thermo Fisher Scientific Inc., Waltham, MA, USA), cDNAs were synthesized using random primers, and real-time quantitative PCR was performed as described above. In the real-time quantitative PCR reactions, ISG15, ISG56, OAS, Mx1, and PKR mRNAs isolated from the transfected cells were amplified using the previously reported primers [29]. The endogenous reference gene, glyceraldehyde-3-phosphate dehydrogenase (GAPDH), was included as an internal control. The independent duplicate real-time PCR data of treatments were normalized to that of GAPDH. The relative mRNA expression levels were quantified by the 2^−∆∆CT^ method [30], in which CT is the threshold cycle. The results were reported as the fold-changes of mRNA expression levels relative to those of mock-treated, non-transfected cells.

### 2.10. Molecular Docking and Protein-Ligand Interaction

To determine protein–ligand binding and interactions, the studied FMDV 3C^pro^ structure was modeled using the published three-dimensional (3D) structure of a wide-type 3C^pro^ as the reference [25,31]. Briefly, the FMDV 3C^pro^ structure was generated based on the deduced amino acid sequence of FMDV type A, NP05 (GenBank: MZ923645.1) [25] by homology modeling with the PDB ID: 2WV4.pdb using the SWISS-MODEL [32]. The modeled 3C^pro^ structure quality was validated by quantitative model energy analysis (QMEAN) [33], the QMEANDisCo scoring function [34], and MolProbity [35], respectively. The 3D structures of AGL (CID:5318517), DAG (CID:21679042), and NEO (CID:9848024) were retrieved from the PubChem database in the sdf format. The molecular docking was performed with AutoDock Vina [36] in the PyRx suite version 0.9.8 [37]. The grid box of 22 Å × 22 Å × 22 Å with the grid center at x = 50, y = 50, z = 66 were defined. The molecular docking of the ligands on the FMDV 3C^pro^ structure was conducted as previously described [25,31]. The two-dimensional (2D) structures of protein–ligand interactions were analyzed using BIOVIA Discovery Studio Visualizer (San Diego, CA, USA), and the 3D orientation of the docked complexes was visualized using Chimera v1.16 (San Francisco, CA, USA) [38].

## 3. Results

### 3.1. Cytotoxicity of the Andrographolides on BHK-21 and HEK 293T Cells

The cytotoxicity of AGL, DAG, and NEO was evaluated with a wide range of these compound concentrations using the MTS assay. After incubation with the andrographolides at concentrations up to 150 µM for 24 h, mild and moderate cytotoxic effects of these andrographolides on BHK-21 and HEK 293T cells were observed at 100 and 150 µM, respectively, using an inverted microscope. The CC50 values of AGL, DAG, and NEO tested with HEK 293T cells were higher than those with BHK-21 cells (Table 1), indicating that BHK-21 cells were slightly susceptible to the andrographolides. The CC10 values were also determined as the baseline concentrations of the andrographolides that caused 10% cell death. Therefore, the treatment with a compound at the CC10 concentration would result in at least 90% viable cells compared to the DMSO control. In the following experiments, CC10 values would be used as maximum doses of non-cytotoxic concentrations whenever the specific doses were higher than the CC10.

### 3.2. Antiviral Activities of AGL and DAG after Infection

**Figure 1:**
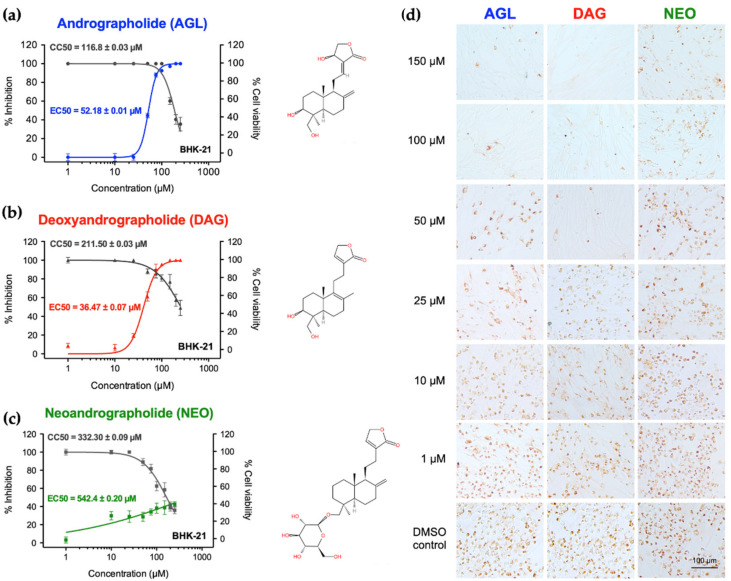
Antiviral activities of AGL, DAG, and NEO against FMDV in BHK-21 cells. (**a**–**c**) Cytotoxicity in BHK-21 cells and FMDV inhibitions by dose-dependent manners were carried out using MTS assay (dark grey) and IPMA (blue for AGL, red for DAG and green for NEO, respectively); (**d**) Antiviral activities of andrographolides were conducted by the presence of the positive FMDV-infected BHK-21 cells in various concentrations compared to that of cell controls with 0.01% DMSO. The data are presented as means and SD from three independent experiments.

**Figure 2:**
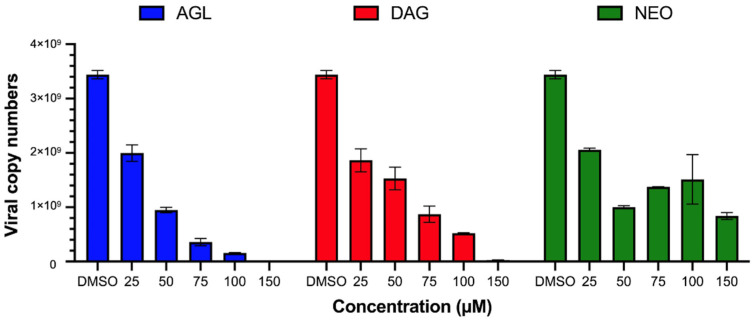
Viral copy numbers in andrographolides treated, FMDV infected BHK-21. Viral nucleic acids from BHK-21 cells infected with FMDV and incubated with different concentrations of AGL, DAG, or NEO were quantified by RT-qPCR. FMDV-infected BHK-21 cells incubated with 0.01% DMSO were served as viral controls. The data are shown as average viral copy numbers and SD from three independent experiments.

The antiviral activity was determined by incubating the andrographolides with BHK-21 cells after 2 h of FMDV infection for 24 h and detecting viral antigens by IPMA. The IPMA results revealed that DAG could inhibit FMDV replication to a greater extent compared to its parental form (AGL), as the numbers of FMDV-infected cells with brown cytoplasmic staining in the wells treated with 50–150 µM DAG were less than in the AGL treated wells (Figure 1). The EC50 value of DAG was 36.47 ± 0.07 µM with a high SI value of 9.22, and AGL-EC50 was 52.18 ± 0.01 µM with an SI value of 2.23 (Table 2). However, NEO had a minimal antiviral effect with the EC50 > 500 µM, as shown in Figure 1 and Table 2. In addition, the viral nucleic acids from intra- and extracellular viruses were quantified after the infected cells were treated with the andrographolides for 24 h. The viral nucleic acids significantly decreased in cells treated with AGL and DAG in a dose-dependent manner, while NEO reduced viral replication to a lesser extent (Figure 2). The results of IPMA and RT-qPCR were consistent with infected cells and viral copy numbers reduced in response to the increasing doses of andrographolides (Figure 1 and Figure 2).

### 3.3. No Direct Effect of AGL and DAG on Extracellular Viruses

To determine whether AGL, DAG, and NEO could exhibit virucidal effects, FMDV and the andrographolides were incubated with BHK-21cells simultaneously for 2 h. For the protection assay, the cells were treated with the andrographolides for 2 h before viral infection. The results showed that these andrographolides had neither extracellular virucidal nor protection activities as they could not inhibit FMDV infection of cells when applied to cells before or during incubation with FMDV (data not shown).

### 3.4. Effects of AGL and DAG on FMDV 3C^pro^

**Figure 3:**
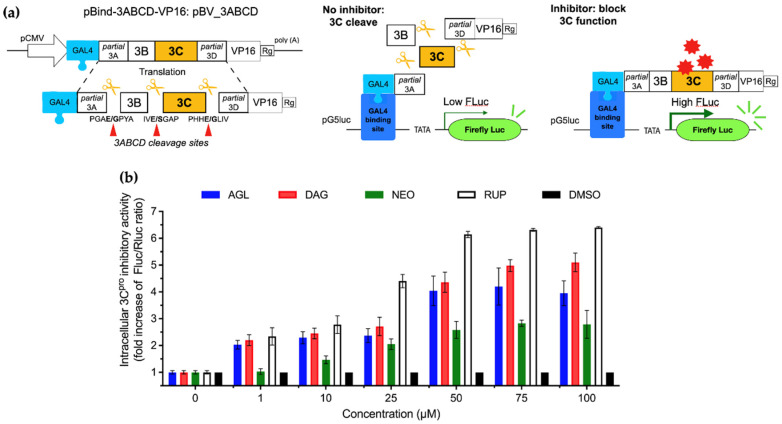
Inhibitory effects of AGL, DAG, and NEO on protease activity of FMDV 3C^pro^ in the intracellular protease assay: (**a**) The schematic diagram of the intracellular protease assay. In this assay, pBV_3ABCD and pG5luc were co-transfected into HEK 293T cells for transient FMDV 3C^pro^ expression. In pBV_3ABCD transfected BHK-21 cells, the active 3C^pro^ cut the polyprotein at the cleavage sites and thus separated VP16 activating domain from Gal4 binding domain. As a result, VP16 was not activated leading to low level of firefly luciferase expression. When andrographolides inhibit protease activity of the 3C^pro^, the polyprotein is not cleaved. Thus, binding of GAL4-binding domain to GAL4 brought the VP16 activation domain in proximity to the VP16 and drove the firefly luciferase expression; (**b**) The protease activities of FMDV 3C^pro^ were determined based on the signal from the firefly luciferase (FLuc) normalized with that of Rinella luciferase (Rluc) from the pG5luc reporter plasmid. The data were presented as the fold increase in FLuc/Rluc ratios from treatments relative to controls (0.01% DMSO). Rupintrivir served as the 3C^pro^ inhibitor control. The experiments were carried out in triplicates, and the data were present as means and SD.

As shown above, AGL and DAG could inhibit viral replication in the post-infection assay; we further investigated whether they could diminish the protease activity of the 3C^pro^ using our intracellular protease assay (Figure 3a, [25]). In this assay, HEK 293T cells were co-transfected with pBV_3ABCD or pBV_mu3ABCD together with pG5luc reporter for 2 h prior to incubating with various concentrations of the andrographolides with the maximum dose of 100 µM. In case the studied dose was greater than the non-cytotoxic concentration (CC10); CC10 was used as an alternative to ensure that the andrographolides had no negative effect on the cells. A schematic diagram of the intracellular protease assay is illustrated in Figure 3a. The protease activity was determined as the inverse correlation of the Fluc/Rluc ratio obtained from the HEK 293T cells transfected with pBV_3ABCD and treated with a drug relative to non-drug control. Without an inhibitor, the 3C^pro^ produced by pBV_3ABCD excised the polyprotein leading to the decreased Fluc signal. When the 3C^pro^ was inhibited by the andrographolides, Fluc was fully activated (details in Materials and Methods). The protease inhibitory effects of DAG and AGL were presented as IC50 and IC90 values (Table 2 and Figure 3b). The IC50 values of DAG and AGL were 25.58 ± 1.41 and 67.43 ± 0.81 µM, respectively. The IC50 value of DAG was slightly lower than its EC50 in the post-infection assay. NEO had a minimal antiviral effect on FMDV; however, NEO lacked anti-3C^pro^ activity. As expected, the cells transfected with pBV_mu3ABCD, which were included as the 3C^pro^ negative control, showed a high Fluc signal irrespective of the addition of andrographolides.

### 3.5. Interfering IFN-Antagonist Activity of FMDV 3C^pro^ by Andrographolides

It has been shown previously that FMDV 3C^pro^ had an IFN antagonist activity which in turn inhibited the expressions of interferon stimulating genes (ISGs) such as IFN-stimulated gene 15 (ISG15), ISG56, 2′,5′-oligoadenylate synthetase (OAS), myxovirus resistance 1 (Mx1), and double-stranded RNA-dependent protein kinase R (PKR) [29]. In this study, we investigated whether the andrographolides reduced the protease activity of FMDV 3C^pro^ and interfered with its IFN-antagonist activity by observing the transcript levels of ISGs. The HEK 293T cells were transfected with pBV_3ABCD for 2 h to allow the 3C^pro^ transient expression, followed by incubating with andrographolides for 24 h and subsequently treated with IFN β for 8 h. The cells were collected for ISG mRNA quantification by RT-qPCR. pBV_mu3ABCD, pBV, and the mock controls were included as 3C^pro^ negative, vector, and non-drug controls, respectively. AGL, DAG, and NEO were used at the concentrations of IC50 and IC90 or CC10 (Table 2). As the IC90 values of AGL and NEO were higher than their CC10, the concentrations of both andrographolides at CC10 were used in the experiments to avoid irregular transfection efficiency among the experimental units caused by compound-induced cell cytotoxicity.

The results demonstrated that ISG mRNA expressions significantly decreased in the IFN β-treated, 3C^pro^ expressing HEK 293T cells compared with the IFN β-treated, non-transfected cells. The empty vector pBV and the full-length protein expression in pBV_mu3ABCD did not contribute to the alteration of ISG mRNA expression levels (Figure S2). Therefore, our experiment confirmed that intracellular 3C^pro^ expressed from pBV_3ABCD suppressed the ISG activation by IFN β as previously reported [29].

**Figure 4:**
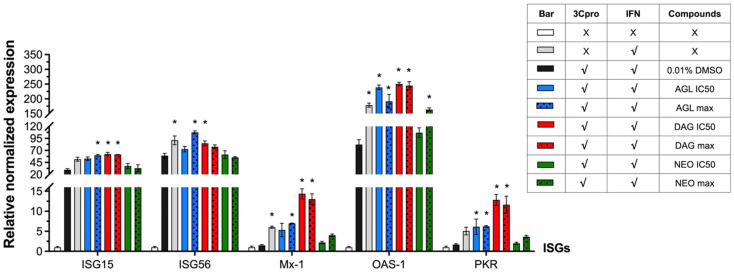
Interference of the andrographolides on inhibition of ISG expression by IFN-antagonist effect of FMDV 3C^pro^ in HEK 293T cells. The cells were transfected with pBV_3ABCD and treated with the compounds at the doses of IC50 and IC90 or CC10. At 24 h after the compound treatments, cells were treated with 1000 IU/mL of IFN β for 8 h. The ISG mRNA levels were analyzed using RT-qPCR. The pBV and pBV_mu3ABCD were included as vector and 3C^pro^ negative controls, respectively (Figure S2). The IFN β-treated, non-transfected cells were used as IFN β control. The data were displayed as the relative ISG mRNA levels of treatments and that of the mock-treated, non-transfected cell control. The experiments were performed in triplicates, and the relative data were presented by means and SD. The asterisks indicate *p*-value < 0.01 compared to the 3C^pro^ expressing cells with 0.01% DMSO.

In the IFN β-treated cells, all of the selected ISG mRNAs, including ISG15, ISG56, Mx-1, OAS-1, and PKR, were activated at high levels (Figure 4). The ISG expression levels significantly decreased in the cells transfected with pBV_3ABCD that provided 3C^pro^ intracellularly. After the 3C^pro^ expressing cells were treated with the andrographolides to inhibit protease activity at their IC50 doses and the maximum doses of the non-cytotoxic concentrations (IC90 or CC10), the suppression of IFN-activated ISG expression by 3C^pro^ was reduced (Figure 4 and Table S1).

The mRNA expression levels of ISG15, ISG56, Mx-1, OAS-1, and PKR were upregulated compared to the non-drug controls (pBV_3ABCD transfected, IFN β treatment, and no drug). Both AGL and DAG could significantly derepressed the IFN-antagonist activity of the 3C^pro^. As expected, NEO, which exhibited minimal antiviral activity and anti-3C^pro^ inhibitory effect, could not reduce the IFN suppression by 3C^pro^ as the ISG mRNA levels in NEO treated cells were not significantly different from those of non-drug controls except OAS-1. The data of ISG mRNA levels of FMDV 3C^pro^ expressing HEK 293T cells, treated with IFN β and incubated with or without andrographolides are presented in Table S1.

### 3.6. Binding Preferences among Andrographolides and FMDV 3^pro^ Active Residues

**Figure 5:**
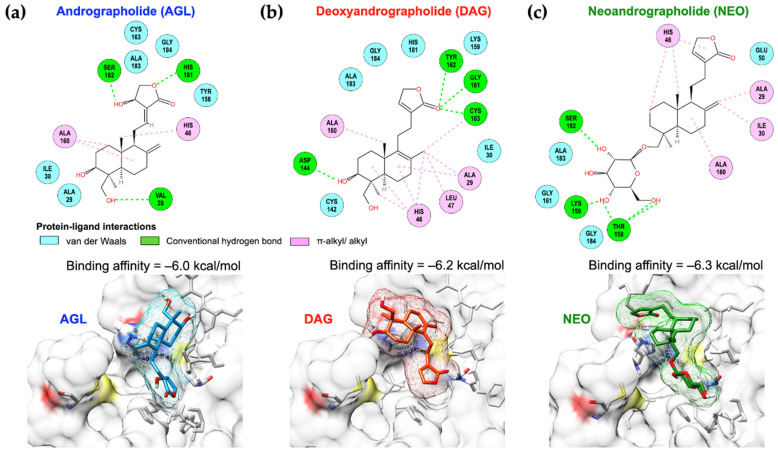
Molecular docking of FMDV 3C^pro^ and protein-ligand interactions of AGL (**a**); DAG (**b**); and NEO (**c**) with the binding pocket of FMDV 3C^pro^ in 2D (upper panel) and 3D structures (lower panel). The binding affinities of AGL, DAG, and NEO are presented on the top of the lower panel, respectively.

To reveal the mechanism of FMDV 3C^pro^ inhibition by the andrographolides, molecular docking was performed to assess the specificity of the andrographolides to the FMDV 3C^pro^ active sides. In silico analysis revealed that AGL, DAG, and NEO could occupy the protease active site of FMDV 3C^pro^ with the binding affinity of −6.0, −6.2, and −6.3 kcal/mol, respectively (Figure 5).

Although the binding affinity of NEO and 3C^pro^ was slightly superior to other andrographolides, it only interacted with an active residue (His46) of the catalytic triad with π-alkyl/alkyl interaction. AGL interacted with both active residues, His46 and Cys163, with alkyl and van der Waals interactions, respectively. DAG preferably reacted to His46 with the bicyclic diterpene, a structured core of DAG, by π-alkyl/alkyl interactions. An oxygen atom of the carbonyl group in the γ-lactone ring of DAG formed an additional H-bond with Cys163 of the 3C^pro^. Moreover, DAG resigned closely to Cys142 residue with a less distance than other andrographolides, which possibly blocked the stability of the 3C^pro^ flap. NEO had a good binding affinity; however, it slightly skewed away from the binding pocket and only reacted to an active residue, His46. Among these andrographolides, DAG presented better binding architectures in terms of the reaction with the active site and a stable residue of the 3C^pro^.

## 4. Discussion

AGL, DAG, and NEO, the main labdane diterpenoid lactone, are bioactive compounds found in *A. paniculate*, a traditional Chinese herb. In this study, we have evaluated their activities on FMDV at various steps of viral infection. Our results suggested that AGL and DAG possessed antiviral activity by reducing FMDV serotype A replication at the post-infection step. This post-entry antiviral effect was consistent with the action of AGL on inhibiting genome replications of hepatitis B virus in HepG 2.2.15 cells with EC50 at 54.10 µM [17], chikungunya virus in HepG2 and BHK-21 cells with EC50 at 77.44 and 88.00 µM, respectively [21], and hepatitis C virus in Huh-7 cells [18]. On the other hand, we have shown that these andrographolides had no antiviral prophylactic activities. This finding corresponded to the minute effect of andrographolides on pre-treatment of influenza virus H9N2, H5N1, and H1N1 with AGL prior to infection in a mouse model [16]. In addition, AGL lacked a virucidal effect on dengue virus infection [39] and could not inhibit EV-D68 attachment or entry [15]. Furthermore, both AGL and DAG failed to protect cells from herpes simplex virus (HSV)-1 infection during viral entry [19] but could inhibit HSV-1 replication in Vero cells by 50% plaque formation with an IC50 of 7.97 µM [40]. However, we found that NEO had no antiviral activity in this study.

The mechanisms by which the andrographolides inhibit viral infections and replications may involve interfering with the regular biological activities of cells or counteracting the crucial mediators of specific intracellular signaling pathways [13]. It has been shown earlier that AGL impeded enterovirus replication by interfering acidification of late endosomes [15]. Additionally, AGL and its derivatives could interact directly with viral components to exert its antiviral activities. For example, AGL could inhibit NS3/4A protease of hepatitis C virus as examined by the cell-based protease activity assay [18]. In addition, the FRET assay revealed that AGL and conjugated AGL formed a covalent bond with 3CL^pro^ from SARS-CoV and SARS-CoV-2 and inhibited the protease activity with IC50 of 5 to 15 µM [41]. However, it is still not known how the andrographolides inhibit FMDV replication.

The 3C protease of FMDV (FMDV 3C^pro^) is a chymotrypsin-like cysteine protease, which is one of the most highly conserved proteins among all picornaviruses, including FMDV [7]. This enzyme plays a crucial role in the viral life cycle by cleaving the picornavirus polyprotein into functional mature structural and non-structural proteins [1,7]. FMDV 3C^pro^ processes 10 of 13 cleavage sites on the polyprotein, making this enzyme an attractive target for antiviral drugs. The active site of FMDV 3C^pro^ contains a chymotrypsin-like catalytic triad composed of Cys163-His46-Asp84 with a Cys142 flap for structural stabilization, which is structurally more specific to FMDV than other picornaviruses [42,43].

To our knowledge, this is the first study on the antiviral and anti-3C^pro^ activity of AGL, DAG, and NEO using the intracellular protease assay. In this assay, the 3C pro was expressed intracellularly by the plasmid pBV_3ABCD transfection. We have shown that AGL and DAG exerted an antiviral activity and inhibited FMDV 3C^pro^ intracellularly in a dose-dependent manner with more potent anti-protease activity for DAG. The structural activity relationships (SARs) of these andrographolides analyzed by molecular docking revealed that the free hydroxyl group (-OH) at C-3 of the lactone ring, found in AGL and DAG but not NEO, could contribute to the increased antiviral activity as reported in hepatitis B and Zika viruses [17,44]. In contrast, the inhibitory effect of AGL and NEO on DNA replication of hepatitis B virus might be subsided by the double bond between C-8 and 17 [17]. These findings confirmed our results that antiviral and anti-3C^pro^ activities of DAG were superior to its parental form and the other derivatives. Furthermore, the basic structure of AGL contains a Michael acceptor group (ketone and also nitro groups), which preferably reacted with the Cys145 residue in the catalytic dyad of SARS-CoV-2 [41]. In addition to the protease action, FMDV 3C^pro^ could suppress the IFN-induced ISG expressions [29]. Thus, we further investigated whether these andrographolides could alleviate the inhibition of ISG mRNA expressions by the 3C^pro^. We found that AGL and DAG could interfere with the IFN-antagonist activity of the 3C^pro^, and thus derepressed the ISG expressions in a dose-dependent manner.

Antiviral agents against FMDV infection could assist other available control measures to confine FMDV spreading and reduce the outbreaks. For instance, T1105, a favipiravir derivative, was an effective FMDV inhibitor, which EC50 was only 12 µM. However, a clinical study showed that a high dose of 200–400 mg/kg/day by oral administration was required to treat an infected FMDV pig [45]. Another antiviral drug, rupintrivir (formerly AG7088), an irreversible 3C protease (3C^pro^) inhibitor, had a limited antiviral effect in a clinical rhinovirus infection in humans [46]. This virus was less susceptible to rupintrivir at high viral passage numbers [47]. Therefore, more potential antiviral agents are still in need.

Plant-based products and extracts, including the isolated compounds (secondary metabolites), have been shown to possess antiviral properties that could suppress several viruses such as influenza viruses, hepatitis C viruses, arthropod-borne flaviviruses, etc. (reviewed in [9]). Some natural compounds could promote effective antiviral environments along various cellular pathways by stimulating the host immune system or exerting cytoprotective properties against oxidative stresses [13]. Some crude extracts from plants showed antiviral effects against FMDV, such as *Morinda elliptica* and *Morinda citrifolia* [48], which contain various bioactive constituents, including anthraquinones, terpenoids, alkaloids, and flavonoids [49]. The antiviral effects of flavonoids have been demonstrated in picornaviruses [50]. For example, luteolin and isoginkgetin had antiviral activity against FMDV by inhibiting the 3C^pro^ [25]. In addition, some natural bioactive compounds that possessed properties following the “Rule of Five” with or without some violations (e.g., molecular weight ≤ 500, Log *p* ≤ 5, H-bond donors ≤ 5, H-bonds acceptors ≤ 10) were potential candidates for antiviral drug development. In regards to the physicochemical properties and ADMET characteristics, including absorption, distribution, metabolism, excretion, and toxicity, the andrographolides in this study passed the Rule of Five with no toxicity.

## 5. Conclusions

FMDV is highly contagious, genetically variable, and quite stable in biological materials. Although vaccination is the most effective tool for disease prevention and control, other measures such as animal movement control, quarantine, and stamping out are also required to stop the outbreak. Herein, we have shown that AGL and DAG impeded FMDV serotype A replication by inhibiting protease and IFN-antagonist activities of FMDV 3C^pro^. These plant-derived antiviral inhibitors have the potential to be used as a supportive tool to the traditional control and prevention strategies for reducing FMD spreads in endemic countries by curtailing the viral spread in the acute phase.

## Figures and Tables

**Table 1:** Cytotoxicity of andrographolide (AGL), deoxyandrographolide (DAG), and neoandrographolide (NEO) in BHK-21 and HEK 293 T cells.

Cytotoxicity (µM)		AGL	DAG	NEO
BHK-21	CC50 ^1^	116.8 ± 0.03	332.3 ± 0.09	211.5 ± 0.03
	CC10 ^2^	94.55 ± 0.02	81.05 ± 0.01	149.94 ± 0.21
HEK 293T	CC50 ^1^	453.00 ± 0.04	651.40 ± 0.32	914.20 ± 0.04
	CC10 ^2^	259.00 ± 0.11	155.55 ± 0.91	270.60 ± 0.03

^1^ CC50: compound concentration required to reduce cell viability by 50% as determined by MTS assay. ^2^ CC10: compound concentration required to reduce cell viability by 10% as determined by MTS assay.

**Table 2:** Effective concentrations (EC50), selective indices (SI), and protease inhibition concentrations (IC50) by intracellular protease assay of AGL, DAG, and NEO.

Andrographolides	IPMA		Intracellular Protease Assay	
	EC50 (µM) ^1^	SI ^2^	IC50 (µM) ^3^	IC90 (µM) ^4^
AGL	52.18 ± 0.01	2.23	67.43± 0.81	267.21 ± 2.43
				(CC10 ^5^; 259.00 ± 0.11)
DAG	36.47 ± 0.07	9.11	25.58 ± 1.41	122.88 ± 2.09
				(CC10; 155.55 ± 0.91)
NEO	542.40 ± 0.20	0.38	111.4 ± 2.04	635.76 ± 2.90
				(CC10; 270 ± 0.03)

^1^ EC50: compound concentration required to effectively protect 50% of cells from virus infection in BHK-21 cells. ^2^ Selective indices: a ratio between cytotoxicity and antiviral activity obtained from dividing CC50 by EC50 (CC50/EC50). ^3^ IC50: compound concentration required to inhibit 50% of intracellular protease activity of FMDV 3C^pro^ in HEK 293T cells. ^4^ IC90: compound concentration required to inhibit 90% of intracellular protease activity of FMDV 3C^pro^ in HEK 293T cells. ^5^ CC10: compound concentration that 90% of HEK 293T cells were viable, and it was used as maximum doses of non-cytotoxic concentrations for intracellular protease activity in HEK 293T cells. Values represent the means ± SD from three independent experiments.

## Data Availability

No new data were created or analyzed in this study. Data is available upon requested.

## Supplementary Materials

The following supporting information can be downloaded at: https://www.mdpi.com/article/10.3390/ani12151995/s1, Figure S1. The optimal time point for detecting ISG mRNA transcript levels in IFN β treated HEK 293T cells in this study. The ISG transcript levels were downregulated from 0 to 2 h, and gradually increased from 4 to 24 h. At the 8 h, the significantly high ISG expressions in IFN β-treated cells were observed using real-time qPCR; Figure S2. The mRNA expression levels of the interferon-stimulated genes (ISGs) in HEK 293T cells transfected with the empty vector pBV or pBV_mu3ABCD. pBV_mu3ABCD expressed the full-length 3B and inactive 3C^pro^ (3C^pro-^). The data presents as means and SD, Table S1. ISG mRNA expression of HEK 293T cells transfected with FMDV 3C^pro^ compared to mock controls.
