# Supplementary material for: Andrographolide and Deoxyandrographolide Inhibit Protease and IFN-Antagonist Activities of Foot-and-Mouth Disease Virus 3Cpro
Source: Animals (Basel). 2022 Aug 7;12(15):1995. doi: 10.3390/ani12151995 (PMC9367459; doi:10.3390/ani12151995)
Supplement: Supplementary file 1 [file animals-12-01995-s001.zip › animals-1814463-supplementary.pdf]

Supplementary data

Supplementary figures

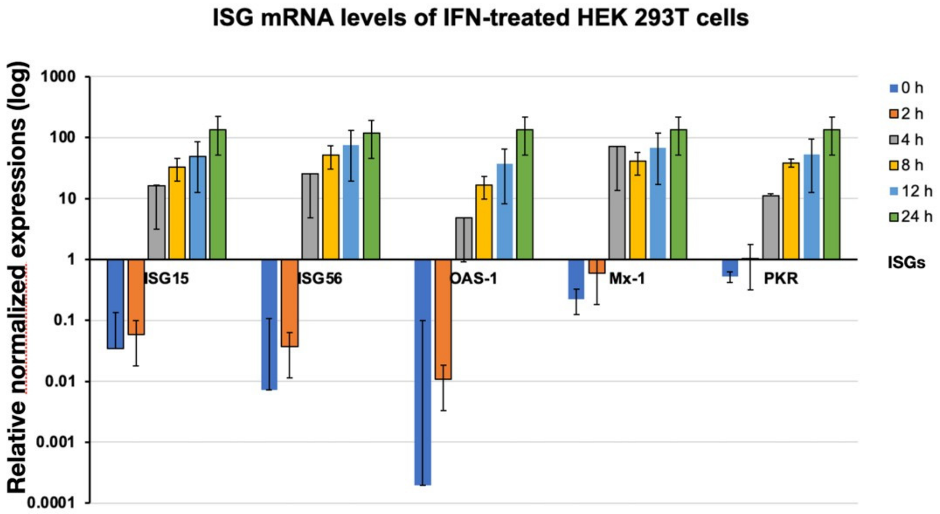

Figure S1. The optimal time point for detecting ISG mRNA transcript levels in IFN-β treated HEK 293T cells in this study. The ISG transcript levels were down-regulated from 0 to 2 h, and gradually increased from 4 to 24 h. At the 8 h, the significantly high ISG expressions in IFN-treated cells were observed using real-time qPCR.

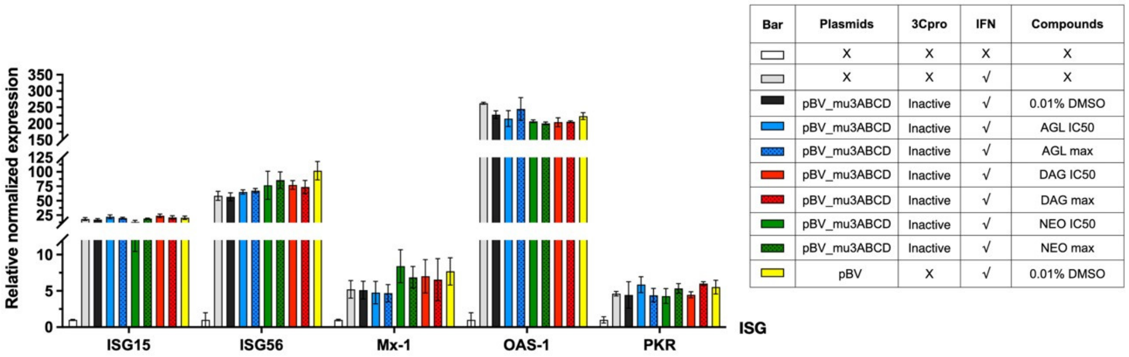

Bar	Plasmids	3Cpro	IFN	Compounds
	X	X	X	X
	X	X	√	X
	pBV_mu3ABCD	Inactive	√	0.01% DMSO
	pBV_mu3ABCD	Inactive	√	AGL IC50
	pBV_mu3ABCD	Inactive	√	AGL max
	pBV_mu3ABCD	Inactive	√	DAG IC50
	pBV_mu3ABCD	Inactive	√	DAG max
	pBV_mu3ABCD	Inactive	√	NEO IC50
	pBV_mu3ABCD	Inactive	√	NEO max
	pBV	X	√	0.01% DMSO

Figure S2. The mRNA expression levels of the Interferon-Stimulated Genes (ISGs) in HEK 293T cells transfected with the empty vector pBV or pBV_mu3ABCD. pBV_mu3ABCD expressed the full-length 3B and inactive $3C^{pro}$ ($3C^{pro}$-). The data presents as means and SD.

Supplementary Table S1. ISG mRNA expression of HEK 293T cells transfected with FMDV 3C^{pro} compared to mock control.

ISG genes	mRNA expression (fold-changes)*							
	mock treated, non-transfected cell	mock treated, transfected cell	AGL		DAG		NEO	
			IC50 dose	Maximum dose	IC50 dose	Maximum dose	IC50 dose	Maximum dose
ISG15	51.39	29.51	52.93	59.99	*62.36*	*64.43*	37.58	32.91
ISG56	90.80	59.05	72.73	107.66	*84.77*	77.60	62.33	55.39
Mx-1	5.99	1.49	5.33	6.98	*14.37*	*13.04*	2.19	4.00
OAS-1	178.30	82.15	*238.78*	191.71	*250.25*	*244.76*	106.88	163.69
PKR	5.02	1.68	6.12	*6.22*	*12.84*	*11.65*	1.99	3.65

*Italics show a significantly difference of ISG mRNA expression in IFN-treated, transfected HEK 293T cells with compounds compared to those 3C^{pro} expressing cells without compounds.
